# Six-Month Synbio^®^ Administration Affects Nutritional and Inflammatory Parameters of Older Adults Included in the PROBIOSENIOR Project

**DOI:** 10.3390/microorganisms11030801

**Published:** 2023-03-21

**Authors:** Chiara Salvesi, Stefania Silvi, Dennis Fiorini, Laura Alessandroni, Gianni Sagratini, Francesco Alessandro Palermo, Renato De Leone, Nadaniela Egidi, Carlo Cifani, Maria Vittoria Micioni Di Bonaventura, Amedeo Amedei, Elena Niccolai, Francesca Scocchera, Fausto Mannucci, Valerio Valeriani, Marco Malavasi, Sara Servili, Andrea Casula, Andrea Cresci, Ivano Corradetti, Maria Magdalena Coman, M. Cristina Verdenelli

**Affiliations:** 1 Scuola di Bioscienze e Medicina Veterinaria, University of Camerino, 62032 Camerino, Italy; 2 Scuola di Scienze e Tecnologie, University of Camerino, 62032 Camerino, Italy; 3 Scuola di Scienze del Farmaco e dei Prodotti della Salute, University of Camerino, 62032 Camerino, Italy; 4 Department of Experimental and Clinical Medicine, University of Florence, 50134 Florence, Italy; 5 COOSS Marche Cooperativa Sociale Onlus, 60121 Ancona, Italy; 6 Labor SPA, 60127 Ancona, Italy; 7 Ambito Territoriale Sociale 16-17-18 Marche Region, 62032 Camerino, Italy; 8 IT Innovative Technology S.r.l., 62032 Camerino, Italy; 9 Fidoka S.r.l., 62020 Ripe San Ginesio, Italy; 10 Caseificio Val D’Apsa & C. Snc, 61029 Urbino, Italy; 11 Synbiofood S.r.l., 62012 Civitanova Marche, Italy; 12 Partner S.r.l., 62078 Spinetoli, Italy; 13 Synbiotec S.r.l., 62032 Camerino, Italy

**Keywords:** probiotics, malnutrition, inflammation, elderly

## Abstract

The physiological changes associated with ageing contribute to the incidence of diseases, morbidity, and mortality. For modern society, it is essential to find solutions to improve elderly people’s health and quality of life. Among promising strategies, the PROBIOSENIOR project proposed a daily six-month supplementation with new probiotic functional foods and nutraceuticals. The aim of this work was to evaluate the modulating effects of the probiotic diet on inflammatory markers and nutritional status. Ninety-seven elderly volunteers were randomly assigned to either a placebo-diet group or a probiotic-diet group (SYNBIO^®^). Faeces, urine, and blood samples were collected before and after the supplementation to determine serum cytokines, biogenic amines, and inflammation markers. Comparing the results obtained before and after the intervention, probiotic supplementations significantly decreased the TNF-α circulating levels and significantly increased those of IGF-1. Biogenic-amine levels showed high variability, with significant variation only for histamine that decreased after the probiotic supplementation. The supplementation influenced the serum concentration of some crucial cytokines (IL-6, IL-8, and MIP-1α) that significantly decreased in the probiotic group. In addition, the Mini Nutritional Assessment questionnaire revealed that the probiotic-supplemented group had a significant improvement in nutritional status. In conclusion, the PROBIOSENIOR project demonstrated how SYNBIO^®^ supplementation may positively influence some nutritional and inflammatory parameters in the elderly.

## 1. Introduction

With improved technologies and advancements in modern medicine, life expectancy is continuously increasing [1]. In this daily challenge, the focus should be to prolong life and also to improve its quality, lowering healthcare costs and social burden. One of the strategies is to identify factors that can improve elderly people’s health, independence, quality of life, and general well-being [2,3,4].

Ageing is a complex phenomenon that results from environmental, genetic, epigenetic, and stochastic events, stimuli in different cell types and tissues, and their interactions throughout life [5]. It is associated with several physiological changes, including metabolic dysregulation, vascular alterations, cognitive decline, and hormone production changes [6,7,8]. There is no clear evidence of which changes are the most significant drivers of the ageing process and how they influence each other, but one of the key mechanisms is surely the inflammation, especially the sterile one (low-grade inflammation) [9].

This phenomenon, called inflammageing, is suggested to be a critical trigger in the pathogenesis of major age-related chronic diseases. This could seriously interfere with a person’s daily life, becoming a significant risk factor for both morbidity and mortality in elderly people [5,9,10,11]. This low-grade, chronic, systemic, inflammatory state is accompanied by immunity dysregulation, leading to an increase in the concentration of pro-inflammatory cytokines [12,13,14]. It is characterized by raised levels of interleukin-1 (IL-1), interleukin-6 (IL-6), and tumour necrosis factor α (TNF-α); all of which have been shown to rise with age and be involved in the pathogenesis of most age-associated diseases [15,16].

Inflammation has been associated not only with an enhanced risk of morbidity, but also with functional decline in older adults, and it has been identified as a key element in the frailty syndrome [17], defined by the decreased functional reserves and increased vulnerability to stressors [18,19]. Strongly connected with frailty is sarcopenia, another common geriatric event characterized by a considerable loss of muscle mass and strength, mobility, and independence [20]. Despite this, the pathogenesis and sarcopenia mechanisms are still poorly understood. Increasing evidence suggests that the age-related effects of the growth hormone IGF-1, androgen, and oestrogen are associated with their incidence and pathogenesis [20,21]. Indeed, in elderly, the variety of hormones promoting the growth of muscle cells, such as testosterone (T), growth hormone (GH), insulin-like growth factor-1, and also mechanical growth factor (MGF), is decreased [22]. Accordingly, previous studies have documented that sarcopenia is associated with decreased IGF-1 signalling, especially the IGF-1 gene-splicing isoform MGF, and the expression of MGF in skeletal muscle cells is decreased in sarcopenia patients [23,24].

Another potential underlying mechanism of sarcopenia could be related to biogenic amines (BAs), since age-related changes in their level have been reported, especially for serotonin and dopamine that vary in ageing [25,26]. Biogenic amines are crucial messengers and regulators of essential cell functions; they act as neurotransmitters, neuromodulators, and neurohormones. Recently, research [27,28] has hypothesized that changes in BA titres may be correlated, in elderly people, with muscles’ weakness that is accompanied by alterations in the architecture of muscular tissue.

Additionally, sarcopenia is strongly related to malnutrition, a well-recognised condition among the elderly, in which dysphagia and oral health problems play a key role, contributing to unwanted weight loss or low body mass index (BMI) [29].

Among promising potential strategies to prevent or reduce the age-associated diseases and disabilities described above, the PROBIOSENIOR project proposed new functional foods and nutraceuticals containing probiotics to improve general well-being and parameters related to ageing and inflammatory processes [30]. The potential beneficial anti-inflammageing effects of a prolonged probiotic-foods-based diet, through the study of high-sensitivity C-reactive protein (hsCRP) levels, gut-microbiota composition, and short-chain-fatty-acid (SCFAs) profile, were already described in Salvesi et al., 2022 [30]. In the present study, we hypothesized that the frailty status in elderly may be correlated to changes of some biological markers that can be positively modulated by a specific probiotic diet. To address this hypothesis, serum cytokines, biogenic amines, and inflammation markers were tested before and after a probiotic-diet administration. An additional and parallel outcome was to assess the nutritional status of the volunteers involved in the study, before and after the dietary intervention.

## 2. Materials and Methods

### 2.1. PROBIOSENIOR Study Design

The PROBIOSENIOR trial has been previously described [30]. It was a randomised, double-blind, placebo-controlled study assessing the effect of daily consumption of the SYNBIO^®^ blend (*Lacticaseibacillus rhamnosus* IMC 501^®^ and *Lacticaseibacillus paracasei* IMC 502^®^/5 billion live cells per daily dose) (SYNBIOTEC Srl, Camerino, Italy), by probiotic-enriched foods or by dietary supplement, on healthy seniors’ status. The study was conducted in accordance with the Helsinki Declaration of 1975, as revised in 1983, and national laws and regulations; the protocol was approved by the Ethics Committee (CERM, Marche, Italy). The participants, recruited in five different boarding homes and several private homes, in the Marche Region (Italy), gave written informed consent for the enrolment. It was planned to include 200 elderly volunteers, but due to Covid emergency, only 97 completed the study. Eligible participants were all healthy male and female seniors, aged > 65 years with hsCRP > 1 mg L^−1^. Exclusion criteria were chemotherapy treatments; severe respiratory, hepatic, and/or renal insufficiency; use of antibiotics in the last week; use of anti-inflammatories in the previous 4 months; and use of probiotic or probiotic-based products in the previous 8 weeks. Eligible participants were randomly assigned to two parallel groups to receive either probiotic-enriched foods and capsules (PROBIOTIC) or the respective placebo (PLACEBO) for 6 months. The randomization was carried out using a computerized randomization list. Six different food products were formulated and used as carriers for delivering probiotic bacterial strains: yoghurt, ‘mozzarella’ cheese, fruit smoothies, ‘ricotta’ cheese, ‘primo sale’ cheese, and chocolate. As an alternative, SYNBIO^®^ in capsules (containing 5 × 10^9^ CFU/capsule) was also provided [30,31]. Volunteers were instructed to take one functional food per day or, in case of impossibility to take the food, a capsule. Urines, faeces, and blood samples were collected before (T0) and the day after the end (T1) of probiotic/placebo supplementation.

### 2.2. Detection and Quantification of Biogenic Amines

Urine was collected in the morning before breakfast in the presence of a nurse, sent to a laboratory within 2 h after collection, and stored at −80 °C until the day of analysis. The preparation of the samples was realized through three phases: deproteinization, derivatization, and purification. BA detection and quantification were performed using a high-performance liquid chromatography (HPLC) Agilent 1260. The chromatographic conditions used were a flow rate of 0.8 mL min^−1^, volume of injection of 20 μL, and column temperature of 40 °C. Samples were separated on a Kinetex C18 2.6 μm 100A 100 × 4.6 mm column. Different mobile phases and gradients were tested to better separate the amines. The HPLC instrument was coupled with an Agilent fluorescence detector. In order to establish the fluorescence-detector (FLD) acquisition wavelength, a multi-excitation analysis and a multi-emission analysis were performed, the first in the 200–400 nm range and the second in the 400–700 nm range. Sensitivity was evaluated by the limit-of-detection (LOD) and limit-of-quantification (LOQ) values. LOD values were from 1.4 μg L^−1^ to 18.75 μg L^−1^, and LOQ values ranged from 5.63 μg L^−1^ to 37.5 μg L^−1^ (Table 1).

### 2.3. Gene Expression and Protein Quantification of TNF-α and IGF-1

Venous blood was collected by qualified nurses. The collection was performed under conditions of fasting, upon overnight resting, in the morning, between 7:00 and 9:00 a.m. Blood was stored in collection vessels with or without anticoagulants, immediately frozen, and properly transferred to a laboratory to be processed and analysed. Peripheral blood was treated to isolate mononucleate cells (PBMC) using the Histopaque-1077 (Sigma-Aldrich, St. Louis, MO, USA), according to the indications of the manufacturer. From the fraction containing the PBMCs, the total RNA was extracted following the protocol provided by the company (Thermo Fisher Scientific, Waltham, MA, USA). The extracted RNA was reverse-transcribed into cDNA using the 5X All-in-One RT MasterMix kit (Invitrogen Life Technologies, Milan, Italy).

For molecular analysis, a quantitative PCR with the SYBR Green method ABI 7300 system (Applied Biosystems) and specific target genes’ primers were used (Table 2). The expression value was normalized, comparing with a housekeeping gene (Glyceraldehyde-3-phosphate dehydrogenase, GAPDH). The results of gene expression were calculated through the cycle-threshold-comparison method (ΔCt = Ct_gene target_ − Ct_GAPDH_). For the measurement of plasma levels of TNF-α and IGF-1, two ELISA kits were used (Human TNF-α and Human IGF-1 ELISA Kit), following the protocols provided by the manufacturer (MyBiosource Inc., San Diego, CA, USA).

### 2.4. Detection and Quantification of Cytokines in Blood and Faeces

To obtain serum, blood was centrifuged at 3000 rpm at 4 °C for 10 min. Serum was collected and stored at −80 °C. Volunteers were asked to collect stool samples after overnight fasting. Each participant received detailed instructions describing the method for material collection. If necessary, the nurse in charge assisted the participant in collecting the sample. A plastic holder was used to collect faeces into a sterilized screw-capped collection container. The inflammatory response was evaluated in serum samples and stool samples of volunteers through the specifically assembled Human Cytokine MAGNETIC Kit for Luminex MAGPIX detection system (Merck, Darmstadt, Germany), following the manufacturers’ instructions. The eleven cytokines evaluated in patients’ serum were granulocyte colony-stimulating factor (G-CSF), interleukin (IL)-1β, IL-2, IL-4, IL-6, IL-8, IL-10, IL-15, IL-17A, macrophage inflammatory protein-1α (MIP-1α), and interferon (IFN)-γ. Moreover, IL-1α, IL-1β, IL-21, IL-22, and interferon-gamma-induced protein 10 (IP-10) were quantified in patients’ stool extract (faecal water). The levels of cytokines were estimated using a 5-parameter polynomial curve. The minimum detection concentrations (MinDC) plus 2 standard deviations (SD) for the evaluated cytokines are reported in Table 3. A value under the MinDC + 2 SD was considered 0 pg mL^−1^.

### 2.5. Mini Nutritional Assessment (MNA)

The Mini Nutritional Assessment (MNA) (Appendix A), designed by the Nestlé company, provides a rapid assessment of nutritional status of elderly patients (65 years of age and older) for identifying malnutrition or the risk of malnutrition [34]. The questionnaire was administered to the volunteers at the beginning of the study and after 6 months of intervention. The questionnaire recording was done with the help of the assigned nurse. The answers to the questionnaires were uploaded by the nurse directly on the data collection platform created specifically for the PROBIOSENIOR project. The MNA questionnaire administered to the volunteers was composed of anthropometric measurements, a global assessment, a dietary questionnaire, and a subjective assessment. Once compiled and uploaded to the platform, the questionnaires were processed by researchers who assigned a score to each answer. The final MNA score characterized elderly patients as well-nourished (MNA ≥ 24), malnourished (MNA < 17), or at risk for malnutrition (MNA 17-23.5) [34].

### 2.6. Statistical Analysis

In the statistical analysis, the one-sample Kolmogorov–Smirnov test was utilized for discriminating between normally and non-normally distributed data. This test is a nonparametric test of the null hypothesis that the population cumulative distribution function (cdf) of the data is equal to the hypothesized cdf. Data were analysed by analysis of variance (ANOVA) between treatment and unpaired *t*-test within T0 and T1. When appropriate, a post hoc analysis was carried out using Tukey’s post hoc test using statistical analysis software (GraphPad Software Inc., San Diego, CA, USA). Comparison of response rates between groups was performed using a chi-squared test. Unless otherwise stated, all assays were performed in either triplicate or three independently repeated experiments, and all the values presented here are means ± standard error of mean (SEM). A value of *p* < 0.05 was considered statistically significant. We noted that no adjustment was made for multiple comparisons, mainly because the present clinical study suffered from major problems related to the COVID-19 emergency. The original study expected the recruitment of 200 subjects, but due to the COVID-19 restrictions, for many subjects recruited and subjected to dietary intervention for 6 months, it was not possible to recover the biological samples, so a lot of data was lost. Furthermore, the amount of acquired data differed for different biological characteristics.

## 3. Results

### 3.1. Detection and Quantification of Biogenic Amines

**Figure 1:**
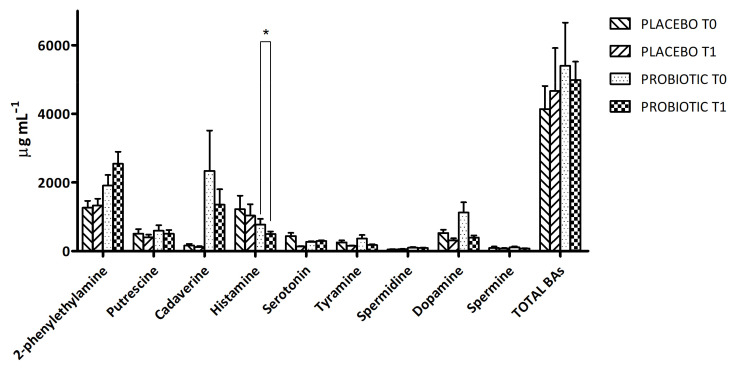
Biogenic-amine (BA) concentration in urine samples of placebo and probiotic-supplemented groups at T0 and T1. The results are expressed as mean values ± SEM (µg mL^−1^). * Significantly different (*p* < 0.05) from T0 by Tukey’s test, one-way ANOVA.

The method used in this study allowed the quantification of 11 BAs at the same time for each urine sample. At T0 and T1, the individual results reported a great inter- and intra-variability, as expected from a biological matrix such as urine. Nine BAs were detected in every analysed sample, while tryptamine and epinephrine could be never detected (hence their data are not shown). In details, Figure 1 shows the BA level at T0 and T1 in the two groups of volunteers reporting the variation in BA total amount, which decreased after probiotic intervention, while increasing in the placebo group. In both cases, the variations were not statistically significant. In general, except for the 2-phenylethylamine that increased in the probiotic group at T1, we observed a decreasing trend in the urinary concentration of BAs in the probiotic group at the end of the supplementation (Figure 1). In particular, a significant decrease of histamine was detected in the probiotic group. Finally, the level of dopamine decreased in the probiotic-supplemented group at T1, while the level of serotonin remained almost stable.

### 3.2. Evaluation of TNF-α and IGF-1

Table 4 shows the mRNA expression of TNF-α and IGF-1 normalized for the housekeeping gene GAPDH, in the probiotic and placebo group. A significantly decreased gene expression was observed at T1 with respect to T0, in both studied groups for TNF-α and only in the probiotic group for IGF-1. Table 4 also shows the serum levels of TNF-α and IGF-1. Despite the typical high standard deviations for TNF-α (due to well-known cytokine inter-individual variability), the data were normally distributed. Concentrations at all time points were distinctly higher than normal (reference range < 20 pg mL^−1^) with mean values > 50 pg mL^−1^. A significant decrease of TNF-α was detected in the probiotic group after supplementation with respect to T0. Moreover, a statistically significant increase in IGF-1 concentration was observed in the probiotic group at T1 with respect to baseline and respect to the placebo group at T1.

### 3.3. Detection and Quantification of Cytokines

#### 3.3.1. Serum Cytokines’ Profile

The serum cytokines profile was evaluated in the two groups of volunteers (placebo and probiotic) before and after the supplementation. The distribution of the cytokines’ majority did not differ significantly after the supplementation in both groups, except for IL-6, IL-8, and MIP-1α, whose levels significantly decreased, after treatment in the probiotic group (Table 5) with respect to T0.

#### 3.3.2. Faeces Cytokines’ Profile

Figure S1 shows the results of the faecal cytokines profile in both the placebo and probiotic volunteers before and after the supplementation. The volunteers’ intestinal cytokines profile did not change after treatment.

### 3.4. Nutritional Status Assessment

The results of the MNA questionnaire at T0 in both groups highlight how a significant percentage of the elderly are malnourished or at risk of malnutrition. Furthermore, the percentage of volunteers with scores falling within the 3 classes (malnourished, at risk of malnutrition, and normal nutritional status) after supplementation is statistically different between the two study arms (chi-square, *p* = 0.001). In particular, as shown in Table 6, in the probiotic-supplemented group, the percentage of volunteers at risk of malnutrition significantly decreased after supplementation, from 54.8% to 35.7%, and the percentage of volunteers with normal nutritional status significantly increased, from 28.6% to 57.1%. The decrease in malnourished subjects should also be emphasized. The placebo-supplemented group had the same percentage of malnourished subjects at T0 and T1, a slight decreased percentage of people at risk of malnutrition, and a slight improvement of those in a normal nutritional status; however, these were not statistically significant.

## 4. Discussion

Ageing is a complex process characterized by physiological decline of biological functions [35], where the inflammation plays a key role in the risk to develop age-related diseases and disabilities. In elderly people, an increase in the low-grade inflammatory markers associated with chronic conditions of ageing has been observed [36]. In addition, malnutrition and sarcopenia are both commonly occurring conditions across older adults. The sarcopenia, when no specific etiologic cause can be identified, is progressive and associated with the aging impact: a reduction in motor neurons; alterations in skeletal muscle tissue, including mitochondrial dysfunction; changes in the hormonal milieu, including a decrease in IGF-1; and an increase in proinflammatory cytokines, such as TNF-α and IL-6. From a pathophysiological point of view, both malnutrition and sarcopenia share many components, a low-inflammatory tone (inflammageing) being an important one.

In this section of the PROBIOSENIOR project, we focused on the gene expression pattern of the two main markers involved in the inflammation process: TNF-α and IGF-1. At the same time, we studied the BA levels, which have recently been discovered to be diagnostic markers of different and impacting diseases [37]. All parameters were evaluated in the elderly volunteers before and after the supplementation to assess the influence of probiotics on these different markers.

At the end of the dietary intervention, interesting findings were observed. Firstly, a significant increase in the circulating level of IGF-1 in the probiotic group, with respect to the placebo-supplemented one, was observed; this positive result is in contrast with the literature, since in elderly, the hormones promoting muscle cell growth, such as GH and IGF-1, generally decrease [22,38]. Moreover, in sarcopenia and frailty conditions, the age-related low signalling of GH, IGF-1, and other hormones are associated with their incidence and pathogenesis [18,21,23]. In addition, recent research has revealed the microbiota role in skeletal growth and homeostasis, and the obtained data, in addition to our previous study [30], support the hypothesis that microbiota modulates circulating levels of IGF-1 in the host by increasing its synthesis [39,40,41]. The SCFAs’ production could be one of the responsible mechanisms by which microbiota increase serum IGF-1. However, the specific pathways by which the microbiota modulates IGF-1 synthesis are still poorly understood, but some potential mechanisms involve SCFA production and the age-related variation in biogenic-amine levels, both of which are study objects in the PROBIOSENIOR project [25,26,27,28,29,30,31,32,33,34,35,36,37,38,39,40,41].

Summarizing, our data suggest that the probiotic supplementation can modulates the GH/IGF-axis, positively bearing on some risk factors of sarcopenia and age-related diseases. Other findings were related to TNF-α levels and its gene expression, whose results were partially influenced by the dietary intervention [42]. A significant decrease in mRNA expression in the probiotic-supplemented group was observed. Accordingly, the circulating TNF-α levels, that normally increase with ageing, in our study significantly decreased in the probiotic group after supplementation with respect to the baseline. Moreover, the same group showed a significant decrease of serum IL-6 after the supplementation. Negative correlations were reported between the rate of protein synthesis in skeletal muscle and levels of C-reactive protein (CRP), IL-6, and TNF-α [41]. High blood TNF-α levels cause an increase in basal energy expenditure, anorexia, and loss of muscle and bone mass in older men and women [42]. The serum cytokine analysis also detected a significant decrease of IL-8 and MIP-1α concentration in the probiotic group after supplementation. MIP-1α and IL-8 are pro-inflammatory chemokines involved in the recruitment of monocytes, macrophages, neutrophils, and other inflammatory cells [43]. In detail, IL-8 is a common chemotactic agent for neutrophils. During the aging process, the function of neutrophils gradually becomes dysregulated and plays an important role in the low-grade chronic inflammation process caused by immune aging and subsequent local tissue damage. A longitudinal study of elderly community-based men and women in the UK also revealed that elevated IL-8 levels are related to a reduction in lower appendicular lean mass and an increased risk of sarcopenia [44]. Although no statistically significant difference was found between the probiotic group and the placebo group with respect to these parameters (TNF-α, IL-6, IL-8, and MIP-1α), the significance within the probiotic group is nonetheless worthy of note and a stimulus for further investigation. Additionally noteworthy is that the lack of between-group significance might also be related to under-powering issues in the current study. In fact, the study was conducted during the COVID-19 pandemic, whose restrictions did not allow us to analyse the expected number of subjects, effectively reducing the possibility of highlighting a significant efficacy of the probiotic compared to the placebo.

The BA levels were also monitored as useful support for clinical diagnosis, and urine is nowadays the main source of BAs available [44] for the analysis thanks to its longer biological half-life in comparison with plasma samples, its inexpensive collection and availability in large quantities, and because the urine tests are non-invasive and simpler than the blood tests. The unbalanced secretion and elimination of pivotal biogenic amines (BAs), such as serotonin (5-hydroxytryptamine, 5-HT), dopamine (DA), norepinephrine (NE), and epinephrine (E), can have severe health consequences since they act not only as neurotransmitters but take part in the regulation of mood, sleep, immune response, thermoregulation, and cardiovascular and gut functions, among others [45]. For many diseases of the central nervous system, such as depression, neurodegenerative diseases (Alzheimer’s disease, Parkinson’s disease), and neurological diseases, a relationship between the level of neurotransmitters and the disease development has been proven. In addition, the aging contributes to the deepening of changes in the secretion of the most important BAs and hormones, which has a significant impact on the development of other diseases, such as cardiovascular disease. Among BAs, the main findings were related to Tyr, DA, and 5-HT. Their balanced levels are important, with octopamine, for proper locomotion [46]. In our study, Tyr levels decreased after the probiotic supplementation, suggesting a beneficial effect, since Tyr concentration is normally lower in healthy volunteers [47]. DA is a neurotransmitter released by the brain that plays several roles in movement, memory, pleasurable reward, behaviour and cognition, attention, inhibition of prolactin production, sleep, mood, and learning. Studies have shown that the DA level is relatively low in healthy people, as opposed to that of patients with some pathologies where it is always found to be high. Previous studies associate increased levels of DA with cigarette smoking [48]. Higher levels of DA in smokers’ subjects than in non-smokers’ urines were reported, so it can be assessed that healthier people present lower DA levels. Positively, in our study 5-HT levels remained mainly stable in the probiotic group and decreased in the placebo one. In general, the variations in BA levels after the dietary intervention were not statistically significant, probably due to the high variability observed in both groups. The only significant result was for histamine, which decreased after probiotic treatment. Maráková and co-workers [47] demonstrate that His concentration is significantly higher in patients with an inflammatory bowel disease (Crohn’s disease) in comparison with healthy subjects, so the statistically significant decrease in the concentrations of His observed in the probiotic-supplemented group underlines the beneficial effects of probiotics on gastrointestinal health.

Overall, the effect of probiotic supplementation was observed on some BAs more than others. However, the detecting limitations and the high variability need additional studies to better investigate the complex pathways behind.

Finally, the biological analyses were supported by the questionnaire survey, which was useful to understand whether the supplementation contributed to improving the general well-being. It is known that older adults are at risk for malnutrition due to physiological, psychological, social, dietary, and environmental risk factors. Weight loss in older adults is often associated with a loss of muscle mass and can ultimately impact functional status. The MNA is a validated scale developed to evaluate the risk of malnutrition in senior citizens and to identify those who might benefit from early intervention [49]. The results of the MNA questionnaire revealed that the probiotic-supplemented group had a statistically significant reduction of volunteers in the “at risk of malnutrition” subgroup and also a decrease in the malnourished percentage at T1 with respect to the placebo group. Malnutrition is prevalent in elderly patients in hospitals, care homes, and those who live in the community. It can lead to various health consequences, such as impaired immune response and reduced muscle strength and weakness, which can lead to an increased risk of complications in patients. For the elderly age group, this can have a knock-on effect on their physical function, mobility, and independence, resulting in a decreased quality of life. The results of this study suggest that SYNBIO^®^ supplementation could be a simple and effective means of achieving some improvements of elderly health.

## 5. Conclusions

In conclusion, taking together the PROBIOSENIOR project results, we highlight how a prolonged diet enriched with SYNBIO^®^ probiotic functional foods and/or dietary supplements is able to counteract the inflammatory process in elderly people through the modulation of inflammatory and nutritional parameters. Given the existence of an inverse relationship between inflammation and muscle strength/power, it is possible to speculate that prolonged SYNBIO^®^ supplementation could be useful in improving the grading of malnutrition and sarcopenia. Further studies with more subjects involved and with targeted parameters are needed to clarify this relationship.

## Figures and Tables

**Table 1:** Limit of quantification (LOQ) and limit of detection (LOD) for each biogenic amine analysed.

Biogenic Amines (BAs)	LOQ (µg L^−1^)	LOD (µg L^−1^)
*Putrescine (Put)*	7.50	2.81
*Cadaverine (Cad)*	5.63	2.81
*Spermidine (Spd)*	7.50	2.81
*Spermine (Spm)*	5.63	2.81
*2-Phenylethylamine (Pea)*	7.50	5.63
*Histamine (His)*	37.50	9.38
*Serotonin (5-HT)*	37.50	9.38
*Tyramine (Tyr)*	9.38	7.50
*Dopamine (DA)*	75.00	37.50
*Tryptamine (Try)*	9.38	5.63
*Epinephrine (E)*	37.50	18.75

**Table 2:** List of primers used in the study.

Gene	Primer Sequence (5′-3′)	Product (bp)	Reference
TNF-α	GTCAACCTCCTCTCTGCCATC CAAAGTAGACCTGCCCAGA	188	[32]
IGF-1	CATGTCCTCCTCGCATCTCTA GCAGCACTCATCCACGATA	212	[33]
GAPDH	AAGCTCATTTCCTGGTATGACAA CGTCTTCCTCTTGTGCTCTTGCTGG	126	[32]

**Table 3:** Minimum detection concentrations (MinDC) + 2 standard deviations (SD) for the evaluated cytokines.

Analyte	MinDC + 2SD
IL-1α	4.51
IL-22	13.60
IP-10	4.40
IL-21	6.80
G-CSF	5.66
IFN-γ	1.79
IL-1β	2.13
IL-2	0.41
IL-4	0.38
IL-6	0.20
IL-8	0.42
IL-10	0.93
IL-15	0.92
IL-17A	1.35
MIP-1α	8.87

**Table 4:** Expression levels of TNF-α and IGF-1 genes both as transcript (ΔCt) and protein (pg mL^−1^) levels, before T0 and after T1 placebo/probiotic supplementation.

	PLACEBO		PROBIOTIC		PLACEBO: T0 vs. T1 (*p*-Value)	PROBIOTIC: T0 vs. T1 (*p*-Value)	T1: PLACEBO vs. PROBIOTIC (*p*-Value)
	T0	T1	T0	T1			
TNF-α (Δct)	3.44 ± 1.13	4.28 ± 1.12	2.95 ± 1.27	3.94 ± 1.69	0.01366 *	0.00383 *	0.21686
TNF-α (pg mL^−1^)	57.82 ± 23.52	48.60 ± 19.68	58.15 ± 19.49	46.52 ± 20.85	0.09922	0.00979 *	0.36123
IGF-1 (Δct)	3.11 ± 1.15	3.20 ± 0.99	2.57 ± 1.08	3.05 ± 0.94	0.39388	0.02684 *	0.28737
IGF-1 (pg mL^−1^)	118.31 ± 23.56	121.88 ± 25.68	118.80 ± 35.79	141.09 ± 44.56	0.32885	0.01271 *	0.04523*

* Significantly different (*p* < 0.05) by Tukey’s test, one-way ANOVA.

**Table 5:** Serum cytokines levels (pg mL^−1^) in the two groups of volunteers (placebo and probiotic) before and after the supplementation.

	Placebo		Probiotic	
	T0	T1	T0	T1
G-CSF	56.53 ± 43.34	72.75 ± 60.39	52.09 ± 18.12	59.03 ± 23.99
IFN-γ	3.71 ± 1.32	1.72 ± 0.88	4.12 ± 1.61	4.06 ± 3.49
IL-1β	6.43 ± 2.18	16.82 ± 12.64	9.62 ± 4.19	5.94 ± 2.36
IL-2	0.45 ± 0.20	0.59 ± 0.45	0.39 ± 0.16	0.35 ± 0.19
IL-4	3.53 ± 2.53	4.99 ± 3.61	2.21 ± 0.57	3.04 ± 1.07
IL-6	8.69 ± 1.88	5.78 ± 1.16	8.96 ± 2.72	3.16 ± 0.68 *
IL-8	57.96 ± 25.16	14.96 ± 2.20	66.19 ± 29.33	12.46 ± 1.05 *
IL-10	17.23 ± 11.64	25.00 ± 18.28	11.02 ± 3.86	21.82 ± 7.89
IL-15	9.66 ± 2.27	8.75 ± 2.35	7.78 ± 1.30	6.67 ± 0.66
IL-17A	4.49 ± 1.77	3.35 ± 2.15	5.87 ± 3.91	5.05 ± 2.59
MIP-1α	15.07 ± 5.06	6.75 ± 4.80	9.21 ± 2.87	3.48 ± 1.79 *

Data are presented as means ± SEM; * Significantly different (*p* < 0.05) from T0, by Student’s *t*-test.

**Table 6:** Mini Nutritional Assessment (MNA) and percentage of subjects in the probiotic and placebo groups with MNA score falling into 3 different categories, before and after the 6-month supplementation/intervention.

		PLACEBO T0		PLACEBO T1		PROBIOTIC T0		PROBIOTIC T1 *	
	MNA Score	Mean ± SD	% Subjects	Mean ± SD	% Subjects	Mean ± SD	% Subjects	Mean ± SD	% Subjects
MALNOURISHED	<17	14.85 ± 1.41	25.0	14.50 ± 1.35	25.0	14.21 ± 2.32	16.6	15.67 ± 0.76	7.2
AT RISK OF MALNUTRITION	17–23.5	21.00 ± 1.45	39.3	20.10 ± 1.41	35.7	21.2 ± 1.19	54.8	21.18 ± 1.50	35.7
NORMAL NUTRITIONAL STATUS	>24	25.35 ± 1.23	35.7	25.09 ± 1.18	39.3	26.9 ± 1.29	28.6	24.98 ± 1.42	57.1

* Significantly different compared to PLACEBO, chi-square, *p* = 0.001.

**Figure A1:**
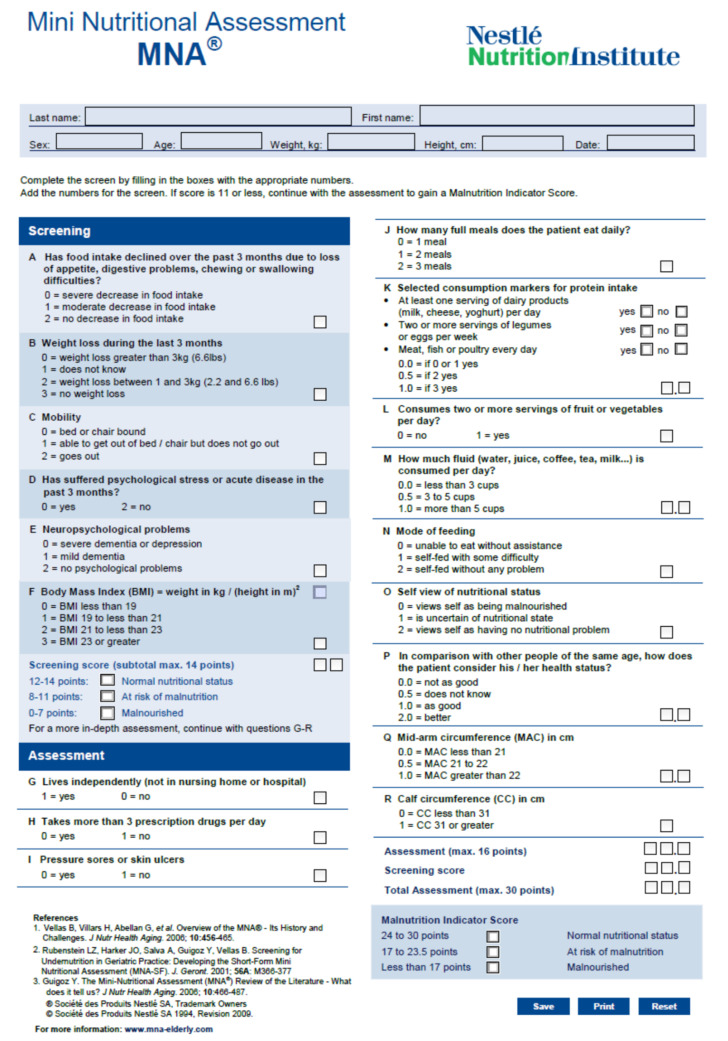
Full-form Nestlé Mini Nutritional Assessment (MNA).

## Data Availability

Not applicable.

## Supplementary Materials

The following supporting information can be downloaded at: https://www.mdpi.com/article/10.3390/microorganisms11030801/s1, Figure S1: Fecal cytokines levels (pg mL^-1^) in the two groups of volunteers (placebo and probiotic), before and after the supplementation (T0 and T1).
