# Supplementary material for: Six-Month Synbio® Administration Affects Nutritional and Inflammatory Parameters of Older Adults Included in the PROBIOSENIOR Project
Source: Microorganisms. 2023 Mar 21;11(3):801. doi: 10.3390/microorganisms11030801 (PMC10053431; doi:10.3390/microorganisms11030801)
Supplement: Supplementary file 1 [file microorganisms-11-00801-s001.zip › microorganisms-2234853-supplementary.pdf]

Supplementary Material

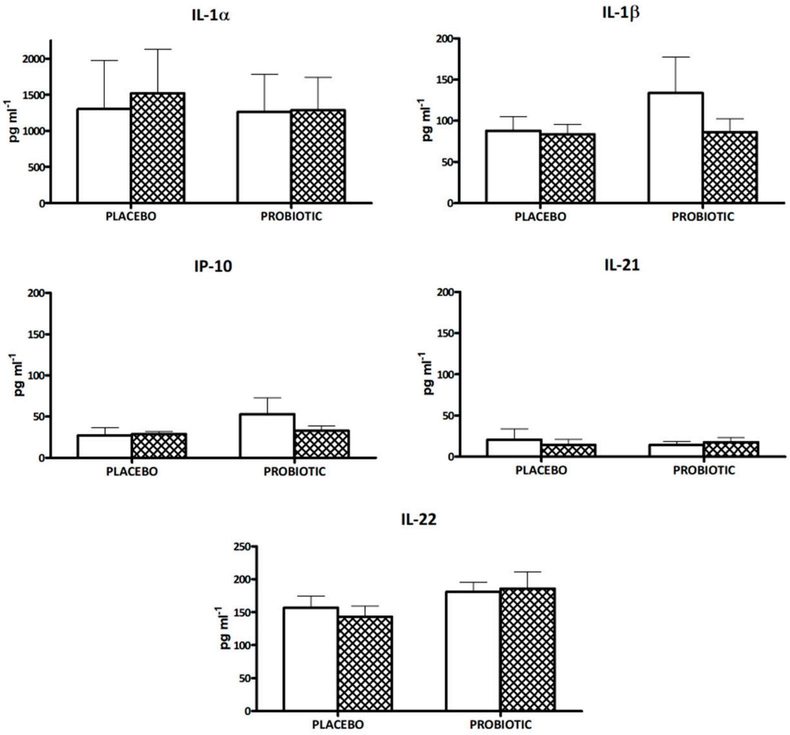

Figure S1. Fecal cytokines levels (pg mL^{-1}) in the two groups of volunteers (placebo and probiotic), before and after the supplementation (□ T0 and ⊠ T1).
